# Is organizational justice climate at the workplace associated with individual-level quality of care and organizational affective commitment? A multi-level, cross-sectional study on dentistry in Sweden

**DOI:** 10.1007/s00420-017-1275-2

**Published:** 2017-11-09

**Authors:** Hanne Berthelsen, Paul Maurice Conway, Thomas Clausen

**Affiliations:** 1 Centre for Work Life and Evaluation Studies (CTA) and the Faculty of Odontology, Malmö University, Malmö, Sweden; 2 Department of Psychology, Copenhagen University, Copenhagen, Denmark; 3 National Research Centre for the Working Environment, Copenhagen, Denmark

**Keywords:** Psychosocial work environment, COPSOQ, Health care, Dentistry, Care quality, Affective organizational commitment

## Abstract

**Purpose:**

The aim of this study is to investigate whether organizational justice climate at the workplace level is associated with individual staff members’ perceptions of care quality and affective commitment to the workplace.

**Methods:**

The study adopts a cross-sectional multi-level design. Data were collected using an electronic survey and a response rate of 75% was obtained. Organizational justice climate and affective commitment to the workplace were measured by items from Copenhagen Psychosocial Questionnaire and quality of care by three self-developed items. Non-managerial staff working at dental clinics with at least five respondents (*n* = 900 from 68 units) was included in analyses. A set of Level-2 random intercept models were built to predict individual-level organizational affective commitment and perceived quality of care from unit-level organizational justice climate, controlling for potential confounding by group size, gender, age, and occupation.

**Results:**

The results of the empty model showed substantial between-unit variation for both affective commitment (ICC-1 = 0.17) and quality of care (ICC-1 = 0.12). The overall results showed that the shared perception of organizational justice climate at the clinical unit level was significantly associated with perceived quality of care and affective commitment to the organization (*p* < 0.001).

**Conclusions:**

Organizational justice climate at work unit level explained all variation in affective commitment among dental clinics and was associated with both the individual staff members’ affective commitment and perceived quality of care. These findings suggest a potential for that addressing organizational justice climate may be a way to promote quality of care and enhancing affective commitment. However, longitudinal studies are needed to support causality in the examined relationships. Intervention research is also recommended to probe the effectiveness of actions increasing unit-level organizational justice climate and test their impact on quality of care and affective commitment.

## Introduction

An important target of research into organizational functioning pertains to identifying factors that may contribute towards enhancing efficiency and quality of production processes, while simultaneously sustaining the motivation and well-being of employees (Morrison et al. 2007). In this perspective, the concept of job resources from the Job Demands-Resources model (Demerouti et al. 2001) acquires particular salience. Job resources touch upon factors in the psychosocial work environment that, on the one hand, enhance the ability of employees to fulfil their work role and achieve work-related goals, and, on the other hand, sustain motivation, well-being, and development of employees (Schaufeli and Bakker 2004).

In many western countries, health care organizations are challenged by changing and increasing needs of the population, costly advances in treatment options, and limited financial resources. Both the European population and the health care workforce are ageing, making the recruitment of new staff a concern for the future (Harford 2009; Rechel et al. 2009; World Health Organization Europe 2015). Staff turnover, recruiting, and introducing new employees is expensive for healthcare institutions, particularly so in the case of highly skilled jobs (Blatter et al. 2012; Li and Jones 2013). These problems are also observable in the Swedish public dental sector, which is characterised by demanding working conditions and an ageing work force and, hence, by projected increases in turnover rates (Bejerot 1998; Berthelsen et al. 2017; Hjalmers 2005; Nyqvist et al. 2016; The National Board of Health and Welfare 2010).

In the present study, we focus on factors in the psychosocial work environment that may be associated with perceived quality of care and affective commitment to the organization, which may play a significant role in work motivation and in retention of staff in dental health care. Being able to deliver care of high quality in health care is important for achieving the intrinsic motivational rewards from the work with patients (Berthelsen et al. 2010; Gunnarsdóttir et al. 2009; Gunnarsdóttir and Rafferty 2006; Hasenfeld 2009) and job satisfaction (Laschinger and Fida 2015; Sasser and Sørensen 2016; Van Eenoo et al. 2016). Both high motivation and job satisfaction may, in turn, promote the retention of healthcare workers (Hayes et al. 2006). Staff’s perception of providing quality care can also be regarded as an indicator of organizational performance, given its significant association with outcomes such as lower staff turnover (Castle and Engberg 2005) and positive patient outcomes (McHugh and Stimpfel 2012). Self-reported perception of care quality at hospitals predicts, for example, patient mortality, failure to rescue, patient satisfaction, and process of care measures (McHugh and Stimpfel 2012). In addition, leadership style, sufficient staffing, and the well-being of staff are related with the quality of care provided (see, e.g., Aiken et al. 2014; Bodenheimer and Sinsky 2014; Castle et al. 2007; Firth-Cozens and Mowbray 2001; Hunt et al. 2014; Laschinger and Leiter 2006; Laschinger et al. 2001, 2014; Li et al. 2013).

Another concept of particular organizational interest in relation to the challenges regarding the recruitment of manpower is affective commitment to the organization, which refers to employees’ identification with and emotional attachment to their workplaces (Meyer and Allen 1997). The previous studies indicate that employees with high levels of affective commitment to their organization are more likely to provide better performances (Sharma and Dhar 2016; Stanley and Meyer 2016) and also to stay in their jobs (Clausen and Borg 2010a). Other studies demonstrate that psychosocial working conditions are associated with affective commitment to the organization (Clausen and Borg 2010b; Meyer et al. 2002). A need of extending the scope of occupational health and safety work by including holistic pro-active strategies has been emphasized, for example, by paying more attention to issues related to leadership and organizational climate (Karanika-Murray and Weyman 2013) and in particular for facing the ageing of the work force (Magnavita 2017).

In the present study, we investigate whether organizational justice climate measured at the level of work-groups is associated with (a) perceived quality of care and (b) affective commitment to the workplace measured at the individual level in the Swedish public dental sector. Organizational justice is a concept that focusses on interpersonal relations in the workplace and whether these relations are handled in a manner that employees perceive as ‘fair’ (Elovainio et al. 2002; Greenberg 1987). Different perspectives of organizational justice exist: Distributive justice dealing with perceived fairness in distribution of, for example, payment, and recognition or tasks; procedural justice taking up the fairness of processes (i.e., whether employees feel that decision-making procedures in the workplace are perceived as fair and transparent), and finally interactional justice, which can be further divided into interpersonal and informational justice (Colquitt et al. 2001; Elovainio et al. 2002). Repeatedly associations have been demonstrated between individual perceptions of organizational justice and various outcomes, such as, for example, organizational citizenship behaviour, performance, job satisfaction, and commitment among employees (Colquitt et al. 2001; Moorman 1991) as well as the health and well-being among employees (Elovainio et al. 2010). However, health care employees collaborate in their daily work, which makes it likely that their behaviours, attitudes, and performances are affected also by the extent to which other employees at their work unit are treated fairly. On the aggregate level, organizational justice can be understood as a climatic factor indicating the extent to which the group as a whole is treated fairly or not; however, the impact of justice at the group level is much less studied than the corresponding construct at the individual level (Naumann and Bennett 2000; Ohana 2014). One of the few studies addressing such impact showed that procedural justice climate could explain unique variance in, for example, helping behaviours at work, but not in affective commitment (Naumann and Bennett 2000). More recent research finds that organizational justice climate can be associated with affective commitment (Ohana 2014). In the context of nursing homes, results from the previous research suggest that shared perceptions of justice and trust may be of greater importance for both work ability and sick leave than individual perceptions (Kiss et al. 2014). Finally, a multi-level study finds a longitudinal association between organizational justice as measured at the level of work-groups and risk of depression among individual employees (Grynderup et al. 2013). Research addressing the relationship between organizational justice climate and care quality is scarce and has so far included only primary care centres (Elovainio et al. 2013; Virtanen et al. 2012). Hypothesis 1 outlines the aims of the present study:

Organizational justice measured at the workplace level is positively associated with affective commitment to the workplace and with self-assessed quality of care measured at the individual level.

To our best knowledge, no previous studies within the context of dentistry have addressed organizational justice, neither as an individual construct nor as an aggregated climate construct. The potential impact of working environment on quality of dental care is a novel field yet to be researched. In addition, affective commitment is under-researched in contrast to concepts such as work engagement and job satisfaction, which often have been addressed in research within the field of dentistry (e.g., Bergström et al. 2010; Buunk-Werkhoven et al. 2014; Denton et al. 2008; Gorter et al. 2008; Hakanen et al. 2008; Harris et al. 2009; Ordell et al. 2013; Schaufeli et al. 2002; Turner et al. 2011).

## Materials and methods

### Study design and participants

Data collection took place at four county councils (regions) of Sweden during the period May 2014 to January 2015. An email including a personal login and password to an online questionnaire was sent to all staff employed at the Public Dental Health Service resulting in an overall response rate of 75% (ranging from 71 to 81% among the regions) after two reminders.

For the present study, we have included non-managerial dental nurses, dental hygienists, and dentists working in general dental practice units with answers from at least five respondents. This resulted in a sample consisting of 900 respondents from 68 units (geographical separate dental practices where people conduct their daily work and share the same local management). The response rate for the chosen subsample was 73%. Almost all respondents had a permanent position (98.1%) and more than half (56%) worked full time there, while only 5% worked half time or less. The characteristics of the study sample are presented in Table 1. Respondents were, on average, 2.5 years older than the non-respondents (*p* ≤ 0.001) and the response rate differed between occupational groups: dentists 64%, dental hygienists 74%, and dental nurses 78% (*p* ≤ 0.001).

**Table 1:** Distribution of baseline characteristics among participants (*n* = 900)

	*n*	%	Mean (SD)
Gender			
Women	832	92.4	
Men	68	7.6	
Age (years)			47.1 (11.9)
Weekly total work hours			36.5 (6.0)
Weekly hours with patient contact			30.1 (9.4)
Job profile			
Dental nurses	499	55.4	
Dental hygienists	196	21.8	
Dentists	205	22.8	
Group size (continuous)			17.3 (7.4)
Group size (categories)			
5–10 respondents	28	41.2	
11–15 respondents	17	25.0	
16–20 respondents	15	22.1	
21–25 respondents	4	5.9	
26–34 respondents	4	5.9	

### Measures

#### Dependent variables

Affective organizational commitment was measured by three items from COPSOQ II (*Would you recommend a good friend to apply for a position at your workplace? Do you feel that your place of work is of great importance to you? How often do you consider looking for work elsewhere?*) (Berthelsen et al. 2014b; Pejtersen et al. 2010). The items were measured by five response options (to a very small extent, to a small extent, to some extent, to a high extent, and to a very high extent), which for analytical purposes were scored 0–25–50–75–100 with 100 indicating the highest degree of commitment.

Quality of care comprises quality of technical care in relation to the interventions intended to promote the patient’s health and quality of interpersonal care including relationships between patients and health professionals (Donabedian 1980). Quality of care was measured by three self-constructed items developed for the purpose of the study: (1) *Are you satisfied with the quality of the work done at your workplace?* and (2) A battery of items: *To what extent do you think that the following issues characterize your ward/department?* (a) *Is the quality of communication with patients good? and* (b) *Is the quality of the actual treatment of patients good?* The first question is more global, while the item on communication represents the interpersonal care and the one on the actual treatment represent the technical aspects. The items were measured by five response options (to a very small extent, to a small extent, to some extent, to a high extent, and to a very high extent), which, for analytical purposes, were scored 0–25–50–75–100. The items were developed and tested as the first part of the Swedish validation project on COPSOQ II; details concerning the procedure have been published previously (Berthelsen et al. 2014a, b, 2016).

For both affective organizational commitment and quality of care, scaling assumptions were examined [e.g., the legitimacy of adding up items to generate scores without weighting or standardization (Likert 1932)] before the scales were established as additive indices with range 0–100. The scale score was set to missing if respondents had answered less than two items. Cronbachs’ alpha was 0.70 for affective organizational commitment and 0.81 for quality of care.

#### Independent variables

Organizational justice is an umbrella construct, covering distributive, procedural, and interactional justice (Cohen-Charash and Spector 2001). Organizational justice was measured by four items from COPSOQ II (*Are conflicts resolved in a fair way? Are employees appreciated when they have done a good job? Are all suggestions from employees treated seriously by the management? Is the work distributed fairly*?). The items were measured by five response options (to a very small extent, to a small extent, to some extent, to a high extent, and to a very high extent), which, for analytical purposes, were scored 0–25–50–75–100 with 100 indicating the highest degree of organizational justice (Berthelsen et al. 2014b; Pejtersen et al. 2010).

As for outcomes, the scale scores were calculated as the mean of the items for each scale, including only those respondents who had answered at least half of the questions included in the scale.

#### Potential confounders

At the unit level (level 2), we controlled for group size, while at the individual level (level 1), we controlled for the demographic variables: gender, age, and occupational group (dental nurses, dental hygienists, and dentists).

### Statistical analysis

The data were analysed using multi-level linear regression analyses. We built two Level-2 random intercept models to examine the associations between group-level organizational justice and the two outcomes separately (organizational affective commitment and quality of care). We computed the intraclass correlation-2 (ICC(2)) to test if there was enough variance shared at the unit level to justify the unit-level mean-aggregation of the individual-level organizational justice scores, using an ICC(2) value of ≥ 0.70 as recommended criterion. The ICC(2) estimates the reliability of the group means and is calculated with the following formula: (Mean Square_(between)_ − Mean Square_(within)_)/Mean Square_(between)_. The independent variables were entered according to a hierarchical procedure. First, we tested an empty model containing only the random intercept (Model 1), which allowed us to compute the ICC(1), i.e., the proportion of variance in the outcome attributable to between-unit effects (Level 2). As a rule of thumb, at least 5% of variance should be at Level 2 to justify the use of multi-level modelling. In Model 2, we then entered all the confounders at both the individual level (Level 1), i.e., gender, age, job profile (dental nurses, dental hygienists, dentists; the latter is the reference category), and the group level (Level 2), i.e., group size (continuous). All the individual-level confounders were grand-mean centred, as recommended (Enders and Tofighi 2007) when a Level-2 independent variable is of substantive interest and the Level-1 independent variables represent nuisance factors that need to be controlled for. In Model 3, we finally entered unit-level organizational justice as Level-2 predictor. Since we aimed to compare nested models, model fit was tested using maximum-likelihood estimation. We compared goodness-of-fit of subsequent models using the deviance statistic -2 log likelihood (-2LL) and the Akaike Information Criterion (AIC). For both indexes, lower scores indicate a better model fit, with AIC also taking model parsimony into account. All analyses were conducted using IBM SPSS for Windows, version 22.0.

## Results

As shown in Table 2, all study variables were significantly correlated in the expected direction. In particular, higher unit-level organizational justice was significantly associated with higher individual-level affective commitment and quality of care.

**Table 2:** Mean, standard deviations (SD), and zero-order Spearman’s correlations between study variables

	Mean	SD	1	2	3
1 Affective commitment	69.9	19.9	–		
2 Quality of care	79.9	14.0	0.45***	–	
3 Organizational justice (group level)	61.0	9.0	0.39**	0.20**	–

***p < 0.001; **p < 0.01

The ICC(2) for organizational justice was 0.76, which indicates sufficient between-unit agreement to justify the mean-aggregation of scores at the unit level. Table 3 shows the results of the two multi-level regression analyses conducted to test the associations between group-level organizational justice and the two individual-level outcomes, i.e., organizational affective commitment and quality of care. The results of Model 1 (model without predictors and confounders) showed substantial between-unit variation for both affective commitment (ICC(1) = 0.17) and quality of care (ICC(1) = 0.12). The confounders entered in Model 2 contributed to explain significant variance in both outcomes (*p* < 0.001), resulting in improved -2LL and AIC indexes pointing to a better model fit. Specifically, both being a dental nurse and a dental hygienist versus being a dentist were related to higher affective commitment and higher quality of care. Higher group size was significantly related to higher affective commitment only. In Model 3, after adjusting for potential confounders, unit-level organizational justice explained additional variance in both outcomes, further improving—2LL and AIC indexes as compared to Model 2. Specifically, for each 10-point increase in unit-level organizational justice, we could observe an approx. 8-point and an approx. 3-point increase in affective commitment and quality of care scores, respectively. Notably, no significant variance in affective commitment was left to be explained after introducing unit-level organizational justice in Model 3.

**Table 3:** Multi-level linear regressions testing the association of organizational justice (each 10-point increase) with affective commitment (*n* = 899) and quality of care (*n* = 900)

	Affective commitment	Quality of care
	B (SE)	B (SE)
Model 1 (empty)		
Unit-level variance (SE)	66.55 (16.60)***	23.80 (9.20)**
ICC(1)	0.17	0.11
− 2LL	7853.01	7329.14
AIC	7859.01	7335.14
Model 2		
Gender (level 1) (ref. male)	0.93 (2.60)	− 0.78 (1.95)
Age (level 1)	0.07 (0.06)	− 0.01 (0.04)
Dental nurse (vs. dentist) (level 1)	5.30 (1.77)**	4.79 (1.32)***
Dental hygienist (vs. dentist) (level 1)	4.90 (1.95)*	3.91 (1.46)**
Group size (continuous) (level 2)	0.38 (0.16)*	0.12 (0.11)
Unit-level variance (SE)	55.23 (14.86)***	22.35 (6.76)**
ICC(1)	0.14	0.11
− 2LL	7829.80	7312.39
AIC	7845.80	7328.39
Model 3		
Gender (level 1) (ref. male)	1.20 (2.54)	− 0.87 (1.87)
Age (level 1)	0.05 (0-05)	− 0.04 (0.04)
Dental nurse (vs. dentist) (level 1)	5.19 (1.73)**	5.20 (1.29)***
Dental hygienist (vs. dentist) (level 1)	4.79 (1.92)*	4.17 (1.41)**
Group size (continuous) (level 2)	0.15 (0.09)	0.03 (0.10)
Organizational justice (level 2)	7.81 (0.69)***	2.71 (0.72)***
Unit-level variance (SE)	2.11 (4.53)	13.93 (5.57)*
ICC(1)	0.006	0.07
− 2LL	7760.43	7299.10
AIC	7778.43	7317.10

***p < 0.001; **p > 0.01; *p > 0.05. *B* unstandardized linear regression coefficient, *SE* standard error, *ICC* intraclass correlation coefficient, − *2LL* − 2 log likelihood, *AIC* Akaike information criterion

## Discussion

The results of this study supported our expectations that higher organizational justice climate at workplaces was associated with higher levels of affective organizational commitment and higher self-assessed quality of care. This corroborates the previous research from primary health care, where procedural justice at the workplace was associated with different aspects of quality care (Elovainio et al. 2013; Virtanen et al. 2012). In addition, our results concerning the importance of organizational justice climate for staff members’ affective commitment were in line with our expectations based on the previous research (cf Ohana 2014). According to Aalto et al. (2014), high levels of organizational justice are associated with high levels of job satisfaction. Other studies indicate that low levels of organizational justice are associated with stress-related symptoms, low psychological well-being (Elovainio et al. 2015), and risk of depression (Grynderup et al. 2013). Taken together, these findings indicate that organizational justice is a job resource that may, on the one hand, contribute to enhancing the well-being of employees, and, on the other hand, support quality and efficiency in the production process.

The previous research in the context of nursing has indicated a mediating effect of affective commitment on the relationship between individual perceptions of organizational justice and work performance (Sharma and Dhar 2016). This corresponds with our finding of a moderate to high bivariate correlation at the individual level between the three main variables under study, and even that the shared perception of justice climate at the workplace was more strongly associated with affective commitment than with quality of care at the individual level. Investigating whether affective commitment mediates the association between organizational justice and perceived quality of care as proposed by Sharma and Dhar (2016) will be a relevant topic for further investigation in a longitudinal design (cf. Taris and Kompier 2006).

The results of our study show that organizational justice climate explained all variation in affective commitment as well as part of the variation in quality of care between units. Affective commitment is an antecedent of actual turnover (Clausen and Borg 2010a) and was also found to reduce the risk of sickness absence (Clausen et al. 2014; Meyer et al. 2002) and poor psychological well-being (Clausen et al. 2015). In a corresponding way, quality of care is associated with a range of patient-related outcomes such as patient satisfaction and patient mortality (McHugh and Stimpfel 2012). Thereby we see a potential in future research aiming at affecting these important outcomes through improvements of organizational justice climate. Even though climatic factors may be complicated to address in interventions, a way forward seems to be through training of managers to promote organizational justice for their subordinates (Nakamura et al. 2016). Though, worth noticing is that this is just one aspect for finding solutions of a complex problem.

Health care of today—including dentistry—is widely organized as team care based on collaboration among different occupations (Kravitz et al. 2015). However, making teamwork working in practice can be challenging (Abelsen and Olsen 2008; Candell and Engstrom 2010). Working in a team is facilitated by a shared understanding of the means and the goals, but perceptions of, for example, efficiency of team care may vary considerably among the dental occupational groups (Muroga et al. 2015). The previous research has also found that dental nurses have a more positive attitude to quality assurance issues and more knowledge on these topics than dentists (Pilgård et al. 2007). On this background, it is worth noticing that the auxiliary staff on average assessed the quality of care to be better than what the dentists did. This result corroborate the previous research pointing to the importance of promoting leadership practices and an organizational climate facilitating teamwork and shared goals in the provision of health care services (Chilcutt 2009; Willcocks 2016).

In the present study, we found that organizational justice climate measured at the workplace level was associated with both individual-level organizational affective commitment and quality of care. These findings support the relevance of multi-level models in investigating complex phenomena in contemporary work organizations. Multi-level models have been used in several studies over the past decade (Bliese and Britt 2001; Clausen et al. 2015; Diez-Roux 2000; Kirwan et al. 2013; Labriola et al. 2006; Li et al. 2013; Nielsen and Daniels 2012; Roux 2004) and must be considered relevant in the study of the current work organizations, as multi-level models (a) offer more realistic analyses of organizational phenomena than analyses that focus exclusively on the individual level and (b) provide knowledge that may be more directly applicable for interventions aiming to improve the psychosocial working conditions in work-groups.

## Limitations and strengths

It can be considered a weakness of the present study that it is based on a cross-sectional study where the same individual rates both the independent and dependent variables. These ratings may, indeed, be affected by variance from unobserved third variables, as, for instance, the mood of the respondent in the response situation. However, the potential risk of common method bias is decreased in a multi-level design (Clausen et al. 2015; Podsakoff et al. 2003). Another limitation may be that the sample comprised one sector only, limiting the generalizability of our findings to other professions within the health care sector. Finally, given the relatively small sample size in the present study, we decided to limit the number of potential confounders to be included in the analyses. On the other hand, we find that the use of items from a well-established questionnaire and a scale measuring quality of care developed from a series of personal interviews constitute strengths of our study as it adds to reliability and construct validity.

In research, organizational justice climate is typically operationalized into sub-dimensions of procedural, distributive, and relational justice (e.g., Ohana (2014). Obviously, this is important for a better understanding of underlying mechanisms. In the present study, we have chosen to operationalize organizational justice climate using a global scale shown to be valid as a group construct and often used for workplace surveys in connection to the organizational development (Berthelsen et al. 2016; Pejtersen et al. 2010). As a considerable proportion of care quality and all the shared variance in affective commitment between units were explained by this operationalization of organizational justice climate, this will facilitate knowledge transferral from research to practice. In addition, the results can be regarded as strengthening the relevance and credibility of the COPSOQ scale for organizational justice for use at an aggregated level.

## Conclusion

Organizational justice climate at work unit level explained all variation in affective commitment among dental clinics and was associated with the individual staff members’ affective commitment and perceived quality of care. These findings suggest a potential for addressing organizational justice climate as a way of promoting quality of care and enhancing affective commitment. However, longitudinal studies are needed to support causality in the examined relationships. Intervention research is also recommended to probe the effectiveness of actions increasing unit-level organizational justice climate and test their impact on quality of care and affective commitment.
